# Think global, act local: Preserving the global commons

**DOI:** 10.1038/srep36079

**Published:** 2016-11-03

**Authors:** Oliver P. Hauser, Achim Hendriks, David G. Rand, Martin A. Nowak

**Affiliations:** 1 Program for Evolutionary Dynamics, Harvard University, Cambridge, MA 02139, USA; 2 Harvard Business School, Boston, MA 02163, USA; 3 Faculty of Business and Economics, University of Osnabrueck, 49069 Osnabrueck, Germany; 4 Department of Psychology, Yale University, New Haven, CT 06520, USA; 5 Department of Economics, Yale University, New Haven, CT 06520, USA; 6 School of Management, Yale University, New Haven, CT 06520, USA.

## Abstract

Preserving global public goods, such as the planet’s ecosystem, depends on large-scale cooperation, which is difficult to achieve because the standard reciprocity mechanisms weaken in large groups. Here we demonstrate a method by which reciprocity *can* maintain cooperation in a large-scale public goods game (PGG). In a first experiment, participants in groups of on average 39 people play one round of a Prisoner’s Dilemma (PD) with their two nearest neighbours on a cyclic network after each PGG round. We observe that people engage in “local-to-global” reciprocity, leveraging local interactions to enforce global cooperation: Participants reduce PD cooperation with neighbours who contribute little in the PGG. In response, low PGG contributors increase their contributions if both neighbours defect in the PD. In a control condition, participants do not know their neighbours’ PGG contribution and thus cannot link play in the PD to the PGG. In the control we observe a sharp decline of cooperation in the PGG, while in the treatment condition global cooperation is maintained. In a second experiment, we demonstrate the scalability of this effect: in a 1,000-person PGG, participants in the treatment condition successfully sustain public contributions. Our findings suggest that this simple “local-to-global” intervention facilitates large-scale cooperation.

Large-scale cooperation is essential to solving many of today’s global problems, such as preserving the rainforest or combating climate change^1^. However, cooperation in the groups is challenging to achieve, because cooperating means to pay a cost to benefit the group as a whole. Thus, everyone in the group is individually better off not contributing, and the “tragedy of the commons” ensues^2^,^3^. To address this collective failure of cooperation, mechanisms have been proposed to promote cooperation in pairwise games or small groups^3^,^4^,^5^. Much less is known, however, about how to maintain cooperation in large groups (which are by definition harder to study in the laboratory). Here we demonstrate a mechanism that can sustain large-scale cooperation.

Experiments focusing on interactions between pairs of people or within small groups (typically, consisting of 3 to 5 people) have established the power of reciprocity for promoting cooperation, be it in the form of repetition^6^,^7^, reputation^8^,^9^,^10^,^11^,^12^, shaming^13^,^14^, network effects^15^,^16^,^17^, threat of expulsion^18^, or costly sanctions^10^,^19^,^20^,^21^,^22^,^23^. The power of reciprocity, however, has been argued to diminish as group size increases, and therefore it seems that these reciprocity-based mechanisms are insufficient for promoting cooperation on a global scale^24^. Although pairs of individuals interacting repeatedly will typically learn to cooperate^6^, even very small groups interacting repeatedly almost always converge on defection^25^.

The reason is that targeted reciprocity is impossible in group interactions: if you stop cooperating towards the group, this harms defectors in your group but also cooperators. The problem can be addressed by adding the opportunity for group members to punish or reward each other based on their contributions^11^,^26^. Such pairwise interactions allow people to target their reciprocity and can stabilise cooperation in small groups. For example, stable cooperation has been observed in studies examining groups of 3 or 4 people in which pairwise interactions occur between all group members^19^,^20^,^21^; and in groups of up to 10 people, so long as group members can sanction at least half of the other group members^27^.

But what about larger groups? Targeted pairwise interactions between most or all group members cannot scale effectively as groups become larger. With increasing group size, it becomes unlikely that a particular group member has the opportunity to interact with any given other member of the group^28^. Thus the settings in which previous experiments have found cooperation to be sustainable, in which group members interact in pairs with a large fraction of other members of the group, is untenable when groups are large.

Does this reasoning imply that reciprocity cannot maintain cooperation in large groups? Here we show that the answer is “no.” We demonstrate that coupling a large repeated group cooperative dilemma to a *sparse* network of repeated pairwise reciprocal interactions averts the “tragedy of the commons,” and sustains cooperation in groups an order of magnitude larger than those studied previously. The number of pairwise interactions need *not* scale with the size of the group: a handful of repeated local interactions can support cooperation on a global scale.

## Methods

To assess the power of such “local-to-global” reciprocity, we developed a novel online software platform called SoPHIE (Software Platform for Human Interaction Experiments, freely available and fully customisable at www.sophielabs.net) to facilitate simultaneous interaction of large numbers of participants^29^. We then used this software to conduct large-scale economic game experiments.

In our first experiment, group sizes were on average 39 people (min = 17, max = 60, sd = 10.28; total *N* = 646), an order of magnitude larger than typical laboratory experiments with 4 players per group^20^,^21^. After providing informed consent, participants played a repeated 2-stage economic game over 20 rounds. In each round of the game, participants first took part in a group contribution stage, and then a pairwise cooperation stage in which they chose actions towards two other group members; for details, see Supplementary Information (SI) Section 1; all experiments were approved by Harvard University Committee on the Use of Human Subjects in Research and carried out in accordance with the relevant guidelines.

**Figure 1:**
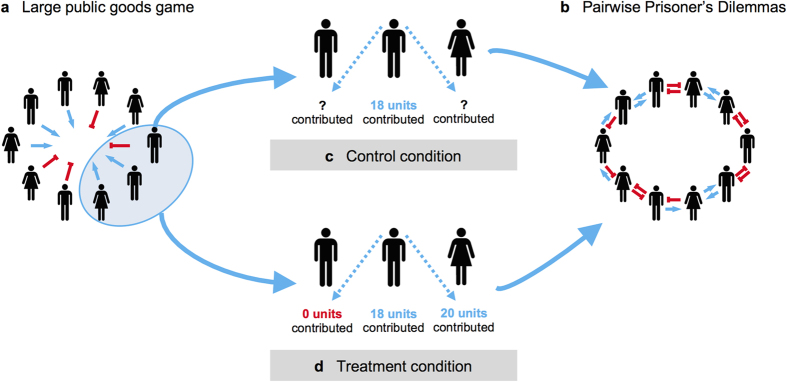
The experimental setup consisted of a series of “global” and “local” interactions. (**a,b**) In each round, participants first took part in a global interaction stage and then in a pairwise local interaction stage. (**a**) In the global stage, groups of on average 39 participants (min = 17, max = 60, sd = 10.28, *N* = 646) played 20 rounds of the Public Goods Game (PGG). In each round, participants were endowed with 20 MUs: they chose how many of these MUs to contribute to a common pool and how many to keep for themselves. The contributed units were doubled and split equally among all group members. (**b)** In the pairwise interaction stage, participants were connected to two other group members on a ring-structured network (in experiment 1; for differences to experiment 2, see SI Section 1). In each round, participants played a Prisoner’s Dilemma (PD) with each neighbour: they could choose to cooperate by paying 6 units to give 18 units to their neighbours; or defect by doing nothing. Thus mutual cooperation yielded a benefit of 12 for both, unilateral cooperation cost cooperators 6 units while providing defectors with 18 units, and mutual defection did not alter the payoff of either participant. (**c,d)** The control and treatment conditions differed in what participants could observe about their neighbours. (**c)** In the control condition, participants were not told how many MUs their neighbours contributed in the PGG stage. (**d)** In the treatment condition, conversely, participants *were* informed of their neighbours’ contributions in the PGG while making their pairwise decisions in the PD.

In the group contribution stage, participants received an endowment of 20 Monetary Units (MUs), and played a public goods game (PGG) with all other members of the group (Fig. 1a). In this global interaction, players chose how many of these MUs to contribute to the public good, and how many to keep for themselves. All contributions were doubled and distributed equally among all group members. Thus contributing benefitted the group as a whole, but was individually costly.

For the pairwise cooperation stage, participants were arranged on a ring-structured network in which they were connected to one neighbour on each side (Fig. 1b). Participants played a separate Prisoner’s Dilemma (PD) game with each of their two neighbours, who remained the same throughout the experiment. In each PD, participants could cooperate by paying 6 MUs to give the other person 18 MUs, or defect by doing nothing. Participants did not have to take the same action towards both neighbours.

Our experiment had two conditions. In the control condition, local-to-global reciprocity was not possible: in the pairwise cooperation stage, participants were not informed about the group contribution behaviour of their neighbours (Fig. 1c). Thus they could not use their pairwise relationships to enforce global cooperation, and we expected group contributions to decrease over time.

In the treatment condition, conversely, participants *were* informed of their neighbours’ group contributions while making their pairwise cooperation decisions (Fig. 1d). Thus local-to-global reciprocity was possible, and we expected that (*i*) subjects would preferentially cooperate in the pairwise stage with neighbours that had contributed larger amounts in the group stage; and (*ii*) as a result, we would observe stable high levels of group contribution (in contrast to the control).

In both conditions, participants were not informed about the total (or average) amount contributed to the PGG across all group members. This lack of PGG information models the fact that in global-level public goods, such as ecosystem conservation, one cannot observe the contribution behaviour of the vast majority of others. Thus, we typically have very little idea of the overall level of public good provisioning.

## Results

**Figure 2:**
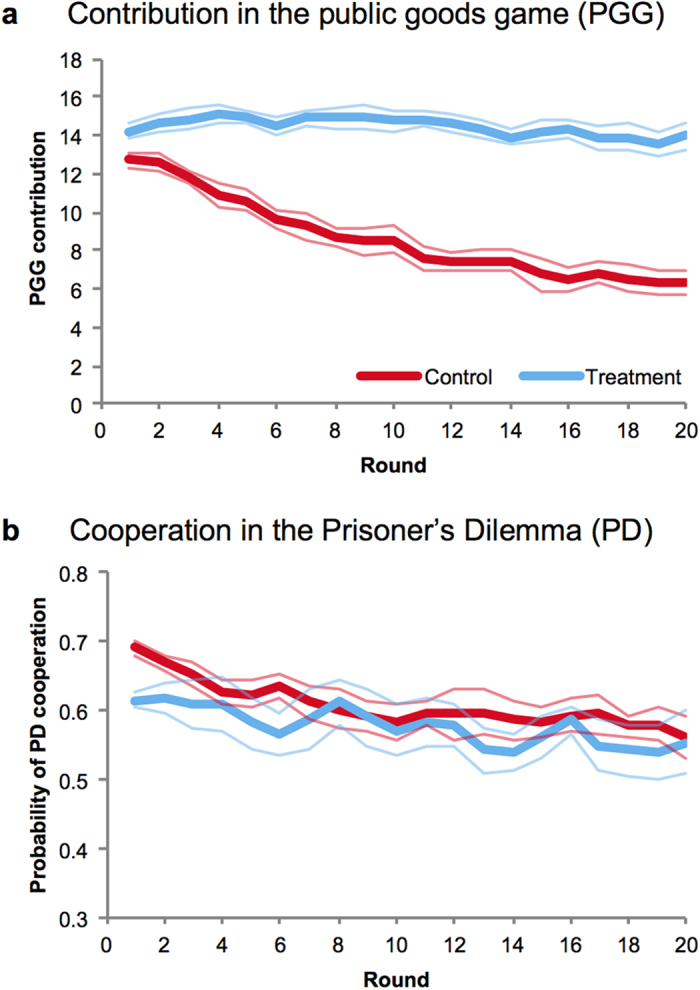
Contributions in the PGG were maintained when participants knew their neighbours’ previous PGG contributions during the pairwise PD stage. (**a**) PGG contributions were maintained at high levels in the treatment condition when participants were informed of their neighbours’ previous PGG contributions. Conversely, in the control condition, the level of contributions in the group cooperation stage decreased quickly over time. (**b**) In the pairwise stage, the level of cooperation did not differ between the control and treatment conditions, but the ways in which the pairwise PDs were used differed substantially (see Fig. 3). (Upper and lower bounds are +/− robust standard errors from the mean clustered on session).

To evaluate these predictions, we began by comparing contributions to the group across our two conditions (Fig. 2a). Indeed, we observed significantly higher average contributions in the treatment compared to the control (coeff = 5.727, *p* < 0.001, Table S1, and Figure S1; all *p*-values generated using linear regression with robust standard errors clustered on session, see SI Section 2 for details). Furthermore, this difference in contribution emerged over time: while participants decreased their contributions from round to round in control (coeff = −0.345, *p* < 0.001), contributions in the treatment were stable (no significant decrease in contribution with round, coeff = −0.051, *p* = 0.098; difference between conditions is significant, as shown by the interaction between round and a dummy for the control treatment: coeff = −0.294, *p* < 0.001, Table S2).

**Figure 3:**
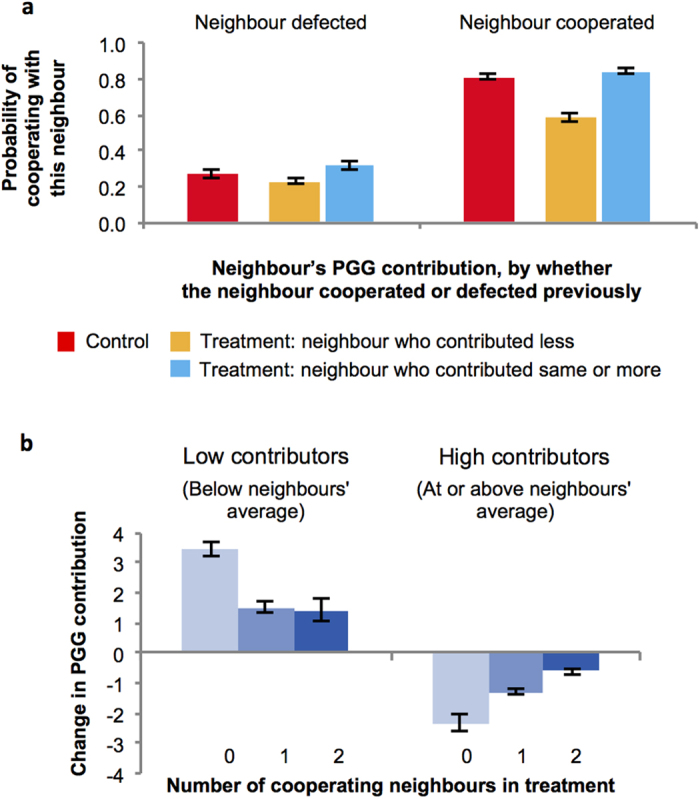
Who gets cooperated with more, or less, in the pairwise PD stage? (**a**) Participants in the treatment condition received less cooperation if they had contributed less than their neighbour, compared to the control group. However, participants did not receive more cooperation than in the control if they contributed at least as much as their neighbour. Thus, local-to-global reciprocity was enacted in local interactions by withholding cooperation from defectors. (**b**) Participants in the treatment condition respond to their neighbours’ decision to cooperate or defect in the pairwise cooperation stage: when both neighbours withheld cooperation from participants who contributed less in the PGG than their neighbours, participants increased their contributions in the PGG in the subsequent round. Conversely, local-to-global reciprocity was buttressing against the temptation to defect: the more PD cooperation high-contributing participants received from their neighbours, the less they decreased their contributions. (Error bars represent robust standard errors clustered on session).

What explains the difference in contribution patterns between treatment and control? Participants’ pairwise cooperation behaviour provides an answer. While there was no significant difference in *average* levels of PD cooperation between conditions (Fig. 2b; coeff = 0.031, *p* = 0.342, Table S3), the specific *ways* that PD cooperation was used did differ importantly (Fig. 3a). In the control, participants were unable to condition their PD cooperation on their neighbours’ PGG contributions (since this information was not available). All they could do was cooperate more with a neighbour who cooperated with them in the previous round (coeff = 0.540, *p* < 0.001, Table S5).

In the treatment, on the other hand, participants took advantage of the contribution information available to them to engage in local-to-global reciprocity. In addition to cooperating more with those who previously cooperated with them in the local PD (coeff = 0.475, *p* < 0.001, Table S5), participants were also more likely to cooperate with neighbours who had contributed at least as much as them in the global PGG (coeff = 0.175, *p* < 0.001, Table S5). Moreover, a significant interaction occurred such that participants were most likely to cooperate with neighbours who cooperated in the PD *and* contributed at least as much as them in the PGG (coeff = 0.168, *p* = 0.002, Table S5). Participants in the treatment condition thus reciprocated not only their neighbour’s previous pairwise cooperation, but also their contributions in the group cooperation stage: they enacted local-to-global reciprocity. This created an incentive to contribute in the PGG that was absent from the control.

There are two ways that this incentive might be used: did participants cooperate more with high contributors, or withhold cooperation from low contributors? To find out, we compared average cooperation rates in control to cooperation rates towards low versus high contributors in treatment (Fig. 3a). If participants were *increasing* cooperation towards high contributors, we would expect cooperation rates towards high contributors in the treatment to be higher than the baseline cooperation rate observed in the control. However, we found no such difference (coeff = 0.037, *p* = 0.303, Table S9). If, on the other hand, participants were *withholding* cooperation from low contributors, we would expect less cooperation towards low contributors in the treatment compared to the control baseline; and this is precisely what we observed (coeff = −0.201, *p* < 0.001, Table S8). Thus we found evidence that local-to-global reciprocity functioned in our experiment via participants withholding cooperation from low contributing neighbours.

Finally, we investigated whether this withholding of cooperation from low contributors was effective in eliciting higher PGG contributions in the next round (Fig. 3b). Interestingly, while receiving PD defection from only one neighbour had no effect on PGG contribution (using number of defecting neighbour as independent variable to predict change in contributions; 1 defecting neighbour: coeff = 0.069, p = 0.871), *both* neighbours defecting in the PD led to a significant increase in PGG contribution in the next round (2 defecting neighbours: coeff = 1.981, p = 0.001, for details see SI Section 2.4). Thus, withholding cooperation was only effective when both neighbours coordinated their withholding.

In addition to disciplining low contributors, PD cooperation also effectively buttressed high contributors against the temptation to reduce their contributions in treatment. High contributors who did not receive cooperation from either neighbour in the PD cooperation substantially reduced their PGG contribution on average. But the more PD cooperation high contributors received from their neighbours, the less this reduction in subsequent PGG contribution occurred (coeff = 0.828, *p* < 0.001, Table S12). Thus we see a full characterisation of the mechanism by which local cooperation stabilised global contribution.

Importantly, these effects were unique to treatment: participant in the control condition did not change their contribution behaviour in response to amount of PD cooperation they received (low contributors: coeff = −0.048, *p* = 0.857, Table S10; high contributors: coeff = 0.253, *p* = 0.183, Table S12), and this differed significantly from what we observed in the treatment (interaction between number of cooperating neighbours and control dummy; low contributors: coeff = 1.105, *p* = 0.002, Table S10; high contributors: coeff = 0.575, *p* = 0.015, Table S12).

**Figure 4:**
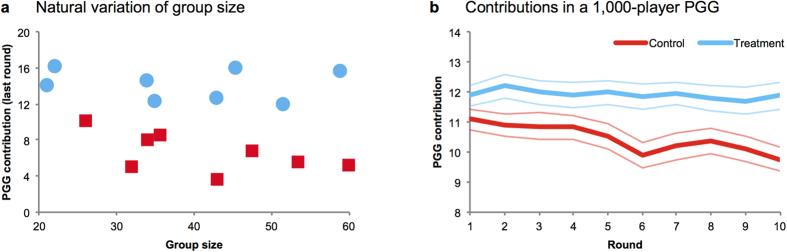
“Local-to-global” reciprocity is invariant to the size of the group. (**a**) We take advantage of random variation across sessions in the number of participants: the size of the group does not have an effect on the level of contributions in the final round of the game in the treatment condition. Indeed, a threefold increase in group size does not affect contributions when “local-to-global” reciprocity is possible. (**b**) In a second experiment (see SI Section 1), we recruited 1,000 participants to play the same large-scale PGG over 10 rounds. Participants in treatment, where “local-to-global” reciprocity with the PD partner was possible, made stable PGG contributions, while participants in control decreased their contributions over time. (Upper and lower bounds are +/− robust standard errors clustered on double-pairs; see SI Section 2 for statistical details).

Finally, we present evidence that the power of local-to-global reciprocity is *scalable*. First, we take advantage of random variation across sessions in the number of participants in the PGG. One might worry that as groups become larger, local interactions with just two neighbours would become less effective at maintaining global cooperation. However, we find no evidence of this (Fig. 4a): a threefold increase in the size of the group had no discernible impact on PGG contributions in the treatment (using group size of each session as independent variable to predict the average contribution in the final round of the game in treatment: coeff = −0.015, *p* = 0.782, Table S13). This lack of relationship suggests the scalability of our intervention, although the small number of independent observations (i.e., groups) prevents this finding from being definitive.

Therefore, we provide further evidence of scalability by conducting a second experiment with a *much* larger PGG group of 1000 people. Participants in the second experiment played a repeated two-stage economic game that was identical to the first experiment, with two exceptions (beyond the larger group size). First, in the pairwise cooperation stage, participants played a PD with just one other member of the group (rather than two others, as in the first experiment). We reduced the number of PD partners to further assess the robustness of our “local-to-global” intervention. Second, we equalised the amount of information participants were given across conditions about the contributions of others: participants in both conditions were informed about the contribution behaviour of one other person each round (their PD partner in the treatment condition, and a random other player in the control condition), unlike in Study 1 where only participants in the treatment condition received information about the contributions of two other players. This change allows Study 2 to demonstrate that the findings of Study 1 were not the result of varying the information provided across conditions. For details on the experimental design of our second study, see SI Section 1.

Despite the extremely large group size of our second experiment, we replicated our earlier results. Average contributions were significantly higher in treatment than in control (coeff = 1.456, *p* = 0.005, Table S15), and this difference emerged over time (interaction between control dummy and round number, coeff = −0.1092, *p* = 0.017, Table S15): while contributions in the treatment were stable (coeff = −0.027, *p* = 0.429), contributions in the control condition decreased with round (coeff = −0.136, *p* < 0.001).

## Discussion

In summary, we have shown that “local-to-global” reciprocity can maintain stable contributions in a large public goods game. Participants withheld cooperation from other group members who contributed less than them. Low contributors, in turn, increased their contributions when their neighbours jointly withheld cooperation from them, while high contributors continued to contribute when their neighbours cooperated with them. Thus, stable levels of contributions emerged in the group cooperation stage of the treatment. In the control, conversely, such local-to-global reciprocity was not possible, and PGG contributions decreased over time.

In his seminal 1968 paper, Garrett Hardin postulated that the “tragedy of the commons” in large populations cannot be solved like any other societal challenge^2^. Thus, Hardin’s summary, “The population has no technical solution; it requires a fundamental extension of morality,” has remained unchallenged. Here we propose the first technical solution to this problem.

Across two experiments, we found that our intervention to sustain large-scale cooperation was not affected by the size of the group: “local-to-global” reciprocity lead contributions in treatment to be sustained in groups several magnitudes larger than previously studied. In particular, targeted reciprocity need not be scaled with the size of the network: instead participants only need to be informed of what a small number of other participants in the network who they interact with did previously, irrespective of the size of the group. The fact that local-to-global reciprocity was effective in such large groups is especially surprising, given that the marginal per capita return of contributing in the PGG becomes smaller as groups grow and thus the incentives to contribute are reduced^30^,^31^,^32^,^33^. However, despite the lower incentives in larger groups, local-to-global reciprocity could maintain cooperation, suggesting that participants were not sensitive to their return from contributing but rather that they wanted to be seen as cooperators. Future research should also explore how similar mechanisms (or other incentives to cooperate^34^,^35^,^36^) could be leveraged to promote cooperation with future generations where there is no possibility of reciprocity and there are no returns from contributing to the public good^37^,^38^, and how stabilising cooperation via local-to-global reciprocity may increase public goods contributions even in situations where no such reciprocity exists via social heuristics and spillover effects 39,40,41,42,43.

Our results may also seem surprising in light of prior findings that PGG contributions could not be sustained even in groups as small as 5 people if participants only had targeted interactions with one other group member^27^. However, these prior findings were generated in a setting where groups were randomly rematched each period specifically to prevent reciprocity effects. Thus we show that when reciprocity is possible, even a very sparse pairwise interaction network can sustain cooperation in very large groups.

Some formal models have suggested that group size poses a challenge for reciprocity-based mechanisms in sustaining cooperation and cannot readily explain the levels of cooperation observed in contemporary and ancient societies^24^. But these models did not consider the possibility of pairwise interactions that allow for targeted action (a possibility which ethnographic research has shown to be a key feature of human interactions in the field^44^). Theory suggest that adding such interactions can stabilise cooperation^11^,^26^. And indeed, our experimental findings demonstrate that reciprocity *can* in fact maintain cooperation in large groups, if each individual has even a very small number of pairwise interactions. Further theoretical work in this vein, for example combining local-to-global reciprocity with models of network structure^45^,^46^,^47^,^48^,^49^, is an important direction for future research.

Our findings build on existing interventions to increase public goods contributions in the real world that have implications for policy-makers^50^,^51^,^52^. Sign-ups among residents in apartment complexes to participate in a voluntary energy reduction program are higher when the sign-up sheet is publicly observable^53^. The more tax evaders are aware that their neighbours know of their delinquency, the higher their compliance with tax repayments^14^. And telling voters that it is possible that they will receive a follow-up phone call to check on their participation increases voter turnout^54^. Our laboratory experiments provide tightly controlled evidence of the mechanism underpinning these field experiment results: when we are provided with information about other people’s cooperative actions, we will reward them for their contributions to our community and to the world at large.

## Additional Information

**How to cite this article**: Hauser, O. P. *et al*. Think global, act local: Preserving the global commons. *Sci. Rep.* **6**, 36079; doi: 10.1038/srep36079 (2016).

**Publisher’s note**: Springer Nature remains neutral with regard to jurisdictional claims in published maps and institutional affiliations.

## Supplementary Material

Supplementary Information
