# Supplementary material for: Think global, act local: Preserving the global commons
Source: Sci Rep. 2016 Nov 3;6:36079. doi: 10.1038/srep36079 (PMC5093714; doi:10.1038/srep36079)
Supplement: Supplementary Information [file srep36079-s1.pdf]

Supplementary Information

for

Think global, act local: Preserving the global commons

Oliver P. Hauser, Achim Hendriks, David G. Rand*, Martin A. Nowak*

Correspondence: martin_nowak@harvard.edu

Table of Contents:

1. Methods ... 2
  1.1 Data collection ... 2
  1.2 Experimental design ... 3
    1.2.1 Experiment 1 ... 3
    1.2.2 Experiment 2 ... 5
2. Statistical details ... 8
  2.1 Group contributions ... 8
  2.2 Pairwise cooperation ... 10
  2.3 Pairwise cooperation strategies ... 12
  2.4 Response in PGG to neighbours' PD choices ... 17
  2.5 Scalability ... 19
    2.5.1 Random variation of group size ... 19
    2.5.2 Large-scale PGG with 1,000 players ... 21
3. Instructions ... 23
  3.1 Experiment 1 ... 23
  3.2 Experiment 2 ... 28
    3.2.1 Control condition ... 28
    3.2.2 Treatment condition ... 33
References ... 37

1. Methods

1.1 Data collection

We recruited U.S. participants for both experiments from the online labour market Amazon Mechanical Turk (AMT). AMT is an online market place in which employers can pay users for completing short tasks – usually referred to as Human Intelligence Tasks (HITs) – for a relatively small pay (generally about $1.00 for 10 minutes of work).

AMT has been shown to be more diverse and more nationally representative than the typical college student sample at major research universities (1-3). Workers who have been recruited on AMT receive a baseline payment and can also be paid a bonus depending on their performance in the task. This setup lends itself well to adopt incentivised economic experiments: the baseline payment acts as the 'show-up' fee and the bonus payment may derive from the workers' behaviour in the economic game and/or other tasks throughout the experiment.

There may, of course, exist potential issues on AMT that would not occur in a traditional laboratory setting. For instance, running an experiment online involves giving up some control over subjects, since they cannot be monitored, as is usually the case in laboratories. That is, it cannot be ruled out that more than a single person is taking part in the experiment or that one person is participating more than once in the experiment (although AMT has put extensive measures into place to avoid this from happening; in addition, we have also implemented ways to carefully screen out any possible re-takers). Finally, the participating subject sample, albeit more diverse and representative than the average college students sample, is biased towards those who participate in online labour markets in the first place. To address these possible concerns, numerous studies have been carried out to validate results collected using AMT (2-4).

Our experiments (described in detail below) were implemented using the interactive experimental platform SoPHIE (Software Platform for Human Interaction Experiments), which is freely available and fully customisable at www.sophielabs.net. (5)

In experiment 1, we recruited a total of 646 participants across 16 sessions. Each session lasted for approximately 35-40 minutes. All participants who completed the experiment earned a $3.00 show-up fee and had the opportunity to earn an additional "bonus" payment depending on their and others' decisions in the public goods game and the prisoner's dilemma. Average earnings from the game including bonus were $4.34.

In experiment 2, a total of 1,352 participants were recruited across 15 sessions. Each session lasted for approximately 15-20 minutes. All participants who completed the experiment earned a $1.00 show-up and could earn a "bonus" payment depending on their decisions and those of other participants in the experiment. Average earnings from the game including bonus were $1.34.

All experiments were approved by Harvard University Committee on the Use of Human Subjects in Research.

1.2 Experimental design

1.2.1 Experiment 1

Participants on AMT joined the experiment by responding to our 'HIT' posted on the AMT website, and being redirected to our external website where the game was hosted. They then received instructions on the experimental game and had to pass a comprehension quiz about the rules of the game (see Section 3.1 for screenshots of instructions and comprehension questions). Participants were not allowed to continue unless they answered all three comprehension questions correctly. Participants were then asked to wait up to 10 minutes for other participants to arrive before the experiment began; once the experiment did begin, all participants started at the same time. A countdown was displayed on their screen for the last three minutes and an audio feedback was played informing them about the remaining time until the experiment would start. Participants who did not respond within 40 seconds after the start of the experiment could not participate in the experiment. All participants were informed upfront that their presence was mandatory to be eligible to take part in this study. AMT workers who had taken part in a previous session of this experiment were not allowed to participate again.

The experiment was conducted one session at a time. For each session, we aimed to maximise the number of participants. The average group size was 39 participants (min = 17, max = 60, sd = 10.28). We launched our experiment only during business hours (9am – 5pm Eastern Standard Time) on weekdays for every session. All participants were assigned to the same condition during a single session. We randomized the order of treatment and control conditions across sessions (8 treatment and 8 control) prior to the start of the experiment.

All participants who were eligible to play (i.e., finished the instructions and the quiz in the allotted time) were arranged in a circular network so that every participant had exactly two neighbours (see Figure 1 of the main text). The network structure did not change over the course of the experiment, except as noted below.

Prior to the beginning of the actual game, all participants took part in a practice round. The practice round was played with two neighbours simulated by a computer, which participants were informed about. The practice round took place simultaneously for all participants to ensure that all participants were paying attention and were ready for the actual game. (See Section 3 for screenshots of the practice rounds.) During the practice round, all times to reach a decision were doubled, from 20 seconds to 40 seconds, to ease familiarisation with the setup.

After the practice round, the real game began with participants interacting with their two neighbours. Due to a technical error, in all sessions neighbours were randomly reassigned after round 1 (but were not informed of this reshuffling). We believe this error is unlikely to have had any long-lasting consequences for our participants; and whatever

consequences it might have had would have worked against our treatment effect, by undermining the power of local-to-global reciprocity after the first round.

From the second round onwards, a participant's neighbours stayed the same as long as neither the participant nor her neighbours dropped out of the game (participant dropouts are a common problem in online studies, unlike in the physical lab, and the solution we take here is standard procedure, see Ref. (6, 7)). Dropouts were eliminated from the circular network and the dropouts' former neighbours were connected. Participants were not told if their neighbour dropped out to avoid a 'restart' effect which has been observed in repeated games (8, 9). Participants were told to pay full attention and to avoid dropping out, or else their payoff—show-up fee and bonus—would be zero.

Since dropouts did occur, one might worry about potential selection effects. Most importantly, there was no difference between the treatment and control in dropout rate (logistic regression using treatment dummy to predict probability of dropout, standard errors clustered on session, $p = 0.752$) or average group size (t-test of group size between conditions using a single indicator variable per condition, $p = 0.690$). Thus, differences in behaviour between the treatment and control cannot be attributed to dropouts. Furthermore, we did not find evidence that the behaviour of dropouts was systematically different from non-dropouts: there was no statistical difference in contributions between dropouts and non-dropouts (linear regression using dropout dummy to predict contributions clustered on session, $p = 0.144$), and contribution amount did not predict the probability of dropping out (logistic regression using contributions to predict likelihood of dropout clustered on session, $p = 0.121$). Unlike in our second experiment (see details below), all of our statistical analyses include non-dropouts and dropouts alike, because the game continued even if a participant dropped out; no participants were excluded from the analysis.

The experiment consisted of a series of 20 rounds. Participants were not told how many rounds they would be playing to avoid potential last-round effects and backwards induction (as in Ref. (10)). Each round was comprised of a public goods game (PGG; stage 1) with all participants in the session contributing to a shared pool, followed by pairwise Prisoner's Dilemmas (PDs; stage 2) between the direct neighbours in the circular network.

In stage 1, participants chose a contribution of between 0 and 20 units in the PGG. All contributions were doubled and every participant in the session received an equal share from the public good. After making their PGG contribution decision, participants in both conditions learned their individual payoff from the PGG. In the treatment condition, the participants were also informed of their neighbours' contributions to the PGG, while participants in the control condition received no additional information.

Across both conditions, participants in stage 2 then played two pairwise PDs with their two neighbours. They could choose between cooperation (paying a cost of 6 units to provide the neighbour with a benefit of 18 units) and defection (paying no cost and providing no benefit). Once all participants had made their choice, in both conditions the

PD actions of the participant's two neighbours were displayed and the participant's payoffs in the current round were summarised. (See Section 3 for instructions and screenshots of the experiment.)

1.2.2 Experiment 2

Participants on AMT joined the experiment by accepting our AMT 'HIT'. They read the instructions (see screenshots in Section 3.2) and had to pass several comprehension questions. Participants waited in an online 'waiting room' for up to 5 minutes for three other participants to arrive. As soon as four participants were ready, the game began immediately. There was no practice round; however, the time to reach a decision in each stage was 10 seconds longer in the first round of the experiment than in later rounds.

The two-stage economic game in the second experiment was similar in many ways to one in the first experiment: participants first made a decision in a group contribution stage—the large-scale repeated PGG—and then in a pairwise cooperation stage—a repeated PD. However, there were also several differences between the two experiments. First, while participants in the first experiment interacted with two players in the PD stage, every participant in experiment 2 played a repeated PD with only one other participant.

Furthermore, participants in this experiment were no longer recruited all at the same time; they were instead recruited in batches of 4 participants, which formed two pairs who played the game at the same time. (We refer to these two simultaneously playing pairs of players as "double pairs.") We required two pairs of participants playing the game simultaneously due to a change to the control condition. Participants in the control in experiment 1 were not informed about anyone's PGG contributions during the PD stage, while participants in the treatment condition always knew the PGG contributions of two other players (those with whom they also played the PD). We argued in experiment 1 that observing the PGG contributions of one's PD partners is crucial to sustaining contributions; however, an alternative "social norm" explanation could be that seeing *anyone else's* PGG contributions is sufficient to maintain contributions (i.e., without playing a repeated PD with that same person). We rule out this "social norm" possibility with a new control condition in experiment 2.

Participants in the control condition in experiment 2 saw the PGG contributions of another player who was part of the larger PGG. This player was *not* the same participant with whom they interacted in the PD stage of the game. They saw instead the contributions of one of the players of the pair that played the game simultaneously, and played a repeated PD with a participant whose contributions they did not see. In the treatment condition, conversely, participants continued to observe the contributions of the player with whom they also played the PD game. Thus, in both conditions, participants saw *someone's* PGG contributions: any difference we observe between control and treatment thus cannot be attributed to a "social norm" of others' contribution, but is caused by interacting directly with the person whose contributions were observable.

(While we required four participants to be playing the game at the same time in the control, we only needed two players at the same time in the treatment condition. To avoid

any differences in decision times or dropout rates between conditions, however, participants in the treatment condition also played the game in batches of 4 participants. Note though that each pair of players played their game independently and was not aware of another pair that played simultaneously.)

Finally, participants in the second experiment did not learn about their payoff from the PGG. Because all 1,000 participants were not online simultaneously, it was not possible to calculate the payoff of each round of the PGG in real time. Participants were told that their earnings from the PGG would be calculated at the end of the study. Thus, participants in neither condition learned whether or not overall levels of contributions in the large group were stable, decreasing, or increasing. The lack of feedback from the PGG implies that conditionally cooperative players (11) would not be able to respond to the changes in contributions by the entire group. However, they would still be able to observe, and respond to, the PGG contributions of the player whose contributions they saw during the PD stage. Furthermore, although the lack of PGG feedback could potentially affect individuals' contribution behaviour, this lack of feedback is the same across both conditions and it could thus not drive any difference between conditions.

The experiment consisted of a series of 10 rounds of the two-stage economic game (as described in more detail above for experiment 1). To avoid end-game effects, participants were not told how many rounds would be played (e.g. see (7)). In stage 1, participants could contribute between 0 and 20 units in the PGG. In stage 2, participants played a PD with another player who remained the same throughout the game: each person could choose to cooperate (paying 12 units to increase the other player's payoff by 36 units) or defect (no cost or benefit to either party) with the other player.

The experiment was conducted one session at a time. For each session, we recruited as many as 200 participants per session, and recruitment continued until we had 500 participants per condition (total N = 1,000) who had completed the game. To keep with random assignment, the order of conditions was alternated across sessions. All participants were assigned to the same condition for every session. In total, we conducted 15 sessions (7 control, 8 treatment) and recruited 1,352 participants, of which 26% of groups did not complete the game due to one or more dropout.

Dropouts in the second experiment were handled differently than in the first experiment. While in experiment 1 a participant who dropped out was simply "replaced" by his or her two nearest neighbours joining the cyclic network and playing the remainder of the game together, this was not possible in experiment 2, as there were only 4 participants in the same stage of the game at the same time. Thus, if one participant dropped out (e.g., by closing his or her Internet browser, or losing Internet connection), the remaining three participants, who were part of the two pairs playing the game simultaneously, could not continue. Although those participants could not finish the game, they were compensated for their time by earning the $1.00 show-up fee and a bonus of $0.30.

Across both conditions, 352 participants (26%) did not complete the game. There was no significant difference in the number of dropout groups between conditions (logistic

regression using treatment dummy to predict probability of dropout at the "double pairs" level, with robust standard errors, $p = 0.714$). Neither did levels of contributions nor rates of pairwise cooperation predict the probability of dropping out (logistic regression to predict probability of dropout, clustered on double pair; using contribution: $p = 0.473$; using cooperation: $p = 0.378$).

Our main analysis focuses on the 1,000 participants (500 per condition) who completed all 10 rounds of the game. However, we find qualitative similar results when dropout groups are included (see Table S13).

2. Statistical details

In experiment 1, all games lasted 20 rounds. In each round of the game, participants had to make three choices. First, how many units they wanted to contribute to a group-wide PGG. Contributions in the PGG are measured on a continuous scale (i.e., integers from 0 to 20 where 0 is full defection and 20 is full cooperation). Then, they made two simultaneous decisions in the PD stage: whether or not to cooperate with each of their two neighbours. Cooperation in the PD is a binary measure (i.e., 1=cooperation, 0=defection).

In experiment 2, all games lasted 10 rounds. In each round of the game, participants made two choices: how many units (between 0 and 20) to contribute in a large-scale PGG and whether or not to cooperate with another participant in the PD.

Unless otherwise indicated, we used linear regression models with robust standard errors clustered on session to account for the fact that decisions of players within a given session are not independent.

2.1 Group contributions

We first asked the basic question of how contributions in the group cooperation stage differed between conditions. We predicted contributions to the public good in the control condition to be less than in the treatment condition. This difference would grow as time passed: participants would maintain stable contributions in the treatment condition, while contributions in the control condition would decrease over time. The dependent variable in our analyses was the amount of units contributed per round. The independent variables were a dummy for the control condition (1=control, 0=treatment) and current round number.

As predicted, we found that participants contributed less on average in the control condition than in the treatment condition, both in the first round (coeff = -1.491, p = 0.018, Table S1 col. 1) and averaged over all rounds (coeff = -5.727, $p < 0.001$, Table S1 col. 2). Furthermore, this difference in contributions emerged over time (interaction between round and control dummy, coeff = -0.294, $p < 0.001$, Table S2 col. 3): we observed a significant decrease in contribution over time in the control (coeff = -0.345, $p < 0.001$, Table S2 col. 1), but not in the treatment (coeff = -0.051, p = 0.098, Table S2 col. 2).

Figure S1 shows the distribution of contributions in the control and treatment conditions across all sessions.

Table S1: Linear regression model estimating the effect of treatment on contributions in the group cooperation stage. The treatment condition is taken as baseline. Standard errors clustered on session.

	First round	All rounds
1=Control	-1.491	-5.727
	(0.561)*	(0.633)***
Constant	14.231	14.484
	(0.413)***	(0.409)***
R^2	0.01	0.12
N	646	11,552

* *p*<0.05; ** *p*<0.01; *** *p*<0.001

Table S2: Linear regression model estimating the effect of round and experimental condition on contributions in the group cooperation stage. In column 3, the treatment condition is taken as baseline. Standard errors clustered on session.

	Control	Treatment	Both
Round	-0.345	-0.051	-0.051
	(0.026)***	(0.027)	(0.026)
1=Control			-2.763
			(0.546)***
1=Control X round			-0.294
			(0.036)***
Constant	12.240	15.003	15.003
	(0.460)***	(0.328)***	(0.317)***
R^2	0.06	0.00	0.15
N	5,981	5,571	11,552

* *p*<0.05; ** *p*<0.01; *** *p*<0.001

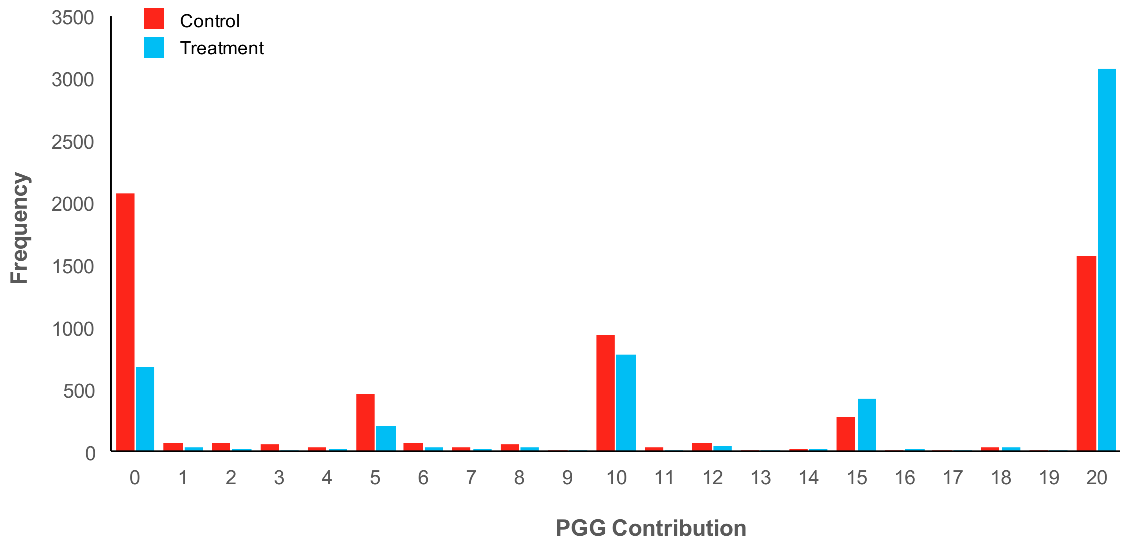

Figure S1. The distribution of the contributions between the control and treatment conditions, pooled across all sessions in experiment 1.

2.2 Pairwise cooperation

We then turned to the question of how participants interacted in the pairwise cooperation stage. Participants could choose to cooperate or defect with each of their neighbours (They did not have to make the same choices for both.)

Here, our unit of observation was the PD cooperation decision (2 observations per participant per round). The independent variable was PD choice (0=defect, 1=cooperate). The dependent variables were a dummy for the control condition (1=control, 0=treatment) and current round number. We use linear regression (despite having a binary DV) in order to have more easily interpretable coefficients; however, we note that using logistic regression instead does not qualitatively change any outcomes.

Although we found that there was significantly more cooperation in the control condition than the treatment in period 1 (coeff = 0.075, $p < 0.001$, Table S3 col. 1), there was no significant difference when considering all rounds (coeff = 0.031, $p = 0.342$, Table S3 col. 2). Furthermore, there was no significant difference between conditions in how cooperation changed over time (interaction between round number and control dummy, coeff = -0.001, $p = 0.492$, Table S4 col. 3): cooperation declined very slightly over time in both the control condition (coeff = -0.005, $p = 0.016$, Table S4 col. 1) and treatment condition (coeff = -0.004, $p = 0.006$, Table S4 col. 2), at a modest rate of on average 0.4% per round.

Table S3: Linear regression model estimating the effect of treatment on levels of cooperation in the pairwise cooperation stage. The treatment condition is taken as baseline. Standard errors clustered on session.

	First round	All rounds
1=Control	0.075	0.031
	(0.015)***	(0.032)
Constant	0.615	0.579
	(0.012)***	(0.028)***
R^2	0.01	0.00
N	1,292	23,104

* p<0.05; ** p<0.01; *** p<0.001

Table S4: Linear regression model estimating the effect of round and experimental condition on PD cooperation. In column 3, the treatment condition is taken as baseline. Standard errors clustered on session.

	Control	Treatment	Both
Round	-0.005	-0.004	-0.004
	(0.002)*	(0.001)**	(0.001)**
1=Control			0.044
			(0.027)
1=Control X round			-0.001
			(0.002)
Constant	0.661	0.617	0.617
	(0.012)***	(0.026)***	(0.025)***
R^2	0.00	0.00	0.00
N	11,962	11,142	23,104

* p<0.05; ** p<0.01; *** p<0.001

2.3 Pairwise cooperation strategies

While the average levels of cooperation in the PD stage did not differ between the two conditions, the *ways* in which the PD was used did differ between conditions.

In the control condition, participants could not condition their behaviour in the PD on their neighbours' PGG contributions, because this information was not available to them. Thus, we only expected participants to condition their PD behaviour on their neighbours' previous cooperation (i.e. to engage in "local" reciprocity). Indeed, we found that participants in the control condition were substantially more likely to cooperate if their partner had cooperated with them in the previous round (using neighbour's action in prior PD round as independent variable with 0=defect, 1=cooperate: coeff = 0.540, $p < 0.001$, Table S5 col. 1).

Conversely, participants in the treatment condition were informed of their neighbours' PGG contributions while making their PD decisions. They were thus able to enact local-to-global reciprocity: they could condition their local PD cooperation with a given neighbour on that neighbour's contribution to the global PGG. Indeed, participants in the treatment were significantly more likely to cooperate with neighbours who were high contributors in the PGG (using neighbour's action in PGG immediately prior to the given PD as independent variable with 0=neighbour contributed less than the participant, 1=neighbour contributed at least as much as the participant, following the definition in (10): coeff = 0.175, $p < 0.001$, Table S5 col. 2).

Participants also engaged in traditional local reciprocity, cooperating more with neighbours who had cooperated with them in the previous PD round (coeff = 0.475, $p < 0.001$, Table S5 col. 2). Furthermore, there was a synergistic interaction between local reciprocity and local-to-global reciprocity (interaction between neighbour's cooperation dummy and neighbour's contribution dummy, coeff = 0.168, $p = 0.002$; Table S5 col. 3), such that participants were most likely to cooperate with neighbours who both cooperated in the previous PD *and* were high contributors in the PGG.

We find qualitatively similar results when we use continuous PGG contribution as the dependent variable in our regression models (Table S6).

Table S5: Linear regression model estimating the effect of a neighbour's previous pairwise cooperation and her group contribution on the participant's willingness to cooperate with her in the current round. Standard errors clustered on session.

	Control	Treatment	Treatment
1=Neighbour cooperated	0.540<br>(0.038)***	0.475<br>(0.014)***	0.356<br>(0.033)***
1=Neighbour contributed same or more than me		0.175<br>(0.021)***	0.088<br>(0.027)*
1=Neighbour cooperated X contributed same or more			0.168<br>(0.035)**
Constant	0.274<br>(0.026)***	0.176<br>(0.012)***	0.233<br>(0.017)***
R^2	0.29	0.27	0.28
N	11,294	10,518	10,518

* *p*<0.05; ** *p*<0.01; *** *p*<0.001

Table S6: Linear regression model estimating the effect of a neighbour's previous pairwise cooperation and her group contribution on the participant's willingness to cooperate with her in the current round. Standard errors clustered on session.

	Control	Treatment	Treatment
1=Neighbour cooperated	0.540<br>(0.038)***	0.411<br>(0.017)***	0.246<br>(0.050)**
1=Neighbour's PGG contribution		0.019<br>(0.001)***	0.014<br>(0.001)***
1=Neighbour cooperated X PGG contribution			0.011<br>(0.003)**
Constant	0.274<br>(0.026)***	0.067<br>(0.011)***	0.124<br>(0.011)***
R^2	0.29	0.31	0.32
N	11,294	10,518	10,518

* *p*<0.05; ** *p*<0.01; *** *p*<0.001

It is possible, however, that participants in the treatment only consulted their neighbour's PGG contribution early in the game, as a way to deciding whether or not their neighbour was likely going to cooperate with them in the PD (rather than using local-to-global reciprocity to enforce PGG contribution). This alternative account suggests that after having played several rounds of the PD with their neighbour, they would then ignore their neighbours' PGG behaviour and only pay attention to their neighbours' last decision in the PD. In that case, the local-to-global effect would disappear in later rounds (the interaction in a similar regression to Table S5 col. 3, for later rounds only, would no longer be significant) because PGG behaviour would only be a diagnostic tool early in the game.

To evaluate this alternative hypothesis, we repeat our analysis above, separately for the first half of the game (rounds 1 through 10) and the second half of the game (rounds 11 through 20) in the treatment condition. Contrary to this alternative explanation, we find that local-to-global reciprocity is enacted in both early and later rounds: participants cooperate more with neighbours who have contributed at least as much as them in the PGG (in early rounds: coeff = 0.192, $p < 0.001$, Table S7 col. 1; in later rounds: coeff = 0.150, $p < 0.001$, Table S7 col. 3), as well as being more likely to cooperate with neighbours who have cooperated with them previously (in early rounds: coeff = 0.380, $p < 0.001$, Table S7 col. 1; in later rounds: coeff = 0.568, $p < 0.001$, Table S7 col. 3),.

In addition, in both early and later rounds, the synergy between PD cooperation and PGG contributions persists: participants are more likely to cooperate with neighbours who have cooperated with them and have also contributed at least as much as themselves in the PGG (interaction between neighbour's previous PD cooperation dummy and neighbour's PGG contribution; in early rounds: coeff = 0.121, $p = 0.004$, Table S7 col. 2; in later rounds: coeff = 0.203, $p = 0.003$, Table S7 col. 3).

Thus, participants consult both their neighbour's previous cooperation in the PD *and* their neighbour's contribution in the PGG in deciding whether to cooperate with them, even in later rounds. Thus it does not seem that participants are using PGG contributions are a diagnostic tool for predicting their neighbours' PD cooperation, but instead are using local-to-global reciprocity in the PD to enforce PGG contribution.

Table S7: Linear regression model estimating the effect of a neighbour's previous pairwise cooperation and her group contribution on the participant's willingness to cooperate with her in the current round, separate for rounds 1-10 (cols. 1 and 2) and for rounds 11-20 (cols. 3 and 4). Standard errors clustered on session.

	Rounds 1-10	Rounds 1-10	Rounds 11-20	Rounds 11-20
1=Neighbour cooperated	0.380<br>(0.013)***	0.292<br>(0.026)***	0.568<br>(0.027)***	0.422<br>(0.049)***
1=Neighbour contributed same or more than me	0.192<br>(0.025)***	0.121<br>(0.030)**	0.150<br>(0.022)***	0.053<br>(0.025)
1=Neighbour cooperated X contributed same or more		0.127<br>(0.030)**		0.203<br>(0.045)**
Constant	0.231<br>(0.022)***	0.278<br>(0.023)***	0.131<br>(0.010)***	0.193<br>(0.016)***
R^2	0.19	0.19	0.37	0.38
N	5,284	5,284	5,234	5,234

* p<0.05; ** p<0.01; *** p<0.001

Two mechanisms could explain our results: either participants cooperated more in the PD with high-contributing neighbours in the PGG, or they cooperated less in the PD with low-contributing neighbours (or both). To find out which mechanism was at work in our data, we compared cooperation rates towards low and high contributors in the treatment condition to cooperation rates in the control condition (where the neighbour's contribution was unknown).

If withholding cooperation of low contributors was occurring, we would expect less PD cooperation with low contributors in the treatment than with unknown contributors in the control; and indeed, this is what we observe (regression including all PD choices from control and PD choices where neighbour contributed less than the participant from the treatment, using treatment dummy as independent variable: coeff = -0.201, $p < 0.001$, Table S8 col. 1; controlling for previous cooperation behaviour does not affect this result, coeff = -0.135, $p < 0.001$, Table S8 col. 2). If more cooperation with high contributors was occurring, conversely, we would expect more PD cooperation with high contributors in the treatment than with unknown contributors in the control; but we find no such effect (regression including all PD choices from control and PD choices where neighbour contributed as much as more than the participant from the treatment, using treatment dummy as independent variable: coeff = 0.037, $p = 0.303$, Table S9).

In short, pairwise cooperation rates in the treatment condition differed towards low-contributing neighbours relative to the control group, but not towards high contributors. Specifically, participants withheld cooperation from low contributors, rather than cooperated more with high contributors.

Table S8: Linear regression model estimating the effects of the treatment on a participant's likelihood of cooperating in the pairwise cooperation stage with a neighbour who contributed *less than* the participant in the group cooperation stage. The baseline group are participants of any contribution level in the control condition. Standard errors clustered on session.

	Neighbour contributed less	Neighbour contributed less
1=Treatment	-0.201<br>(0.026)***	-0.135<br>(0.018)***
1=Neighbour cooperated		0.500<br>(0.034)***
Constant	0.610<br>(0.016)***	0.299<br>(0.024)***
R^2	0.03	0.27
N	15,141	14,270

* p<0.05; ** p<0.01; *** p<0.001

Table S9: Linear regression model estimating the effects of the treatment on a participant's likelihood of cooperating in the pairwise cooperation stage with a neighbour who contributed the *same or more than* the participant in the group cooperation stage. The baseline group are participants of any contribution level in the control condition. Standard errors clustered on session.

	Neighbour contributed same or more	Neighbour contributed same or more
1=Treatment	0.037<br>(0.034)	0.036<br>(0.019)
1=Neighbour cooperated		0.534<br>(0.023)***
Constant	0.610<br>(0.016)***	0.278<br>(0.017)***
R^2	0.00	0.29
N	19,925	18,836

* p<0.05; ** p<0.01; *** p<0.001

2.4 Response in PGG to neighbours' PD choices

We observed that participants in the treatment condition were less likely to cooperate in the PD stage with neighbours who had contributed less than them in the PGG. They withheld cooperation from low contributors. Did this withholding work to elicit more contributions from low contributors in the future?

Indeed, we found that in the treatment, the less a low contributor's neighbours cooperated with her, the more she contributed in the next PGG round (change in contribution predicted by the number of neighbours withholding cooperation (i.e. defecting in PD): coeff = 1.153, $p < 0.001$, Table S10 col. 2). Interestingly, withholding had to be coordinated in order to be effective: having only one neighbour withhold cooperation did not increase subsequent contributions of the low contributor relative to having both neighbours cooperate (coeff = 0.069, $p = 0.871$); it was necessary to have *both* neighbours withhold cooperation in order to motivate low contributors to increase their contributions (0 vs. 2 withholding neighbours: coeff = 1.981, $p = 0.001$) (see Table S11).

Importantly, this effect was unique to the treatment. In the control, having cooperation withheld by one or both neighbours had no effect on low-contributing participants' subsequent PGG contribution (using number of withholding neighbours as independent variable: coeff = 0.048, $p = 0.857$, Table S10 col. 1; using discrete number of neighbours withholding: 0 vs 1 neighbour withholding: coeff = -0.038, $p = 0.920$; 0 vs. 2 cooperating withholding: coeff = 0.129, $p = 0.804$, Table S11 col. 1). Furthermore, when data from both conditions are taken together, a significant interaction between condition and the number of neighbours withholding cooperation demonstrated that the effect of withholding on future contributions was significantly larger in the treatment than the control (interaction between number of cooperating neighbours and treatment dummy: coeff = 1.105, $p = 0.002$, Table S10 col. 3; qualitatively the similar result as describe above using interaction between treatment dummy and discrete number of neighbours, see Table S11 col. 3).

In addition to disciplining low contributors, neighbours' behaviour in PD mechanism also effectively buttressed high contributors against the temptation to reduce contributions in the treatment condition: the more PD cooperation high contributors received from their neighbours, the less they reduced their contributions in the next round (coeff = 0.828, $p < 0.001$, Table S12 col. 2). In the control condition, however, there was no "buttressing effect": receiving more cooperation from neighbours did not protect against declining contributions in control (coeff = 0.253, $p = 0.183$, Table S12 col. 1); an observation that was also confirmed by a significant interaction for the treatment condition only (interacting number of cooperating neighbours with treatment dummy: coeff = 0.575, $p = 0.015$, Table S12 col. 3).

Table S10: Linear regression model estimating the effect of both neighbours' defection in the PD stage on change in contributions of participants who contributed *less than* their neighbours previously in the PGG stage. Standard errors clustered on session.

	Control	Treatment	Both
# Neighbours withholding PD cooperation	0.048<br>(0.255)	1.153<br>(0.183)***	0.048<br>(0.246)
1=Treatment			-0.966<br>(0.369)*
1=Treatment X neighbours withholding			1.105<br>(0.303)**
Constant	1.840<br>(0.247)***	0.874<br>(0.291)*	1.840<br>(0.239)***
R^2	0.00	0.02	0.01
N	2,586	1,663	4,249

* *p*<0.05; ** *p*<0.01; *** *p*<0.001

Table S11: Linear regression model estimating the effect of one or two neighbours' defection in the PD on change in contributions of participants who contributed *less than* their neighbours previously in the PGG. Standard errors clustered on session.

	Control	Treatment	Both
1 neighbour withheld PD cooperation	-0.038<br>(0.365)	0.069<br>(0.411)	-0.038<br>(0.353)
2 neighbours withheld PD cooperation	0.129<br>(0.500)	1.981<br>(0.393)**	0.129<br>(0.483)
1=Treatment			-0.430<br>(0.426)
1=Treatment X 1 neighbour withheld			0.107<br>(0.531)
1=Treatment X 2 neighbours withheld			1.852<br>(0.614)**
Constant	1.869<br>(0.261)***	1.439<br>(0.355)**	1.869<br>(0.252)***
R^2	0.00	0.02	0.01
N	2,586	1,663	4,249

* *p*<0.05; ** *p*<0.01; *** *p*<0.001

Table S12: Linear regression model estimating the effect of both neighbours' cooperation in the PD on change in contributions of participants who contributed the *same or more than* their neighbours previously in the PGG. Standard errors clustered on session.

	Control	Treatment	Both
# Neighbours cooperating in PD	0.253<br>(0.171)	0.828<br>(0.132)***	0.253<br>(0.166)
1=Treatment			0.319<br>(0.290)
1=Treatment X neighbours cooperating			0.575<br>(0.209)*
Constant	-2.523<br>(0.217)***	-2.205<br>(0.208)***	-2.523<br>(0.209)***
R^2	0.00	0.02	0.02
N	3,061	3,596	6,657

* p<0.05; ** p<0.01; *** p<0.001

2.5 Scalability

2.5.1 Random variation of group size

Finally, we present evidence that our "local-to-global" reciprocity is scalable across different sized groups. We take advantage of random variation across sessions in the number of participants in the PGG to illustrate this. One might worry that as groups become larger, local interactions between just two neighbours might be ineffective at stabilising contributions in the global PGG.

However, we find no evidence for this: contributions in the final round of the game do not decline as groups become larger in the treatment condition (coeff = -0.015, p = 0.782, Table S13 col. 2; all regressions in this section take the group as the unit of observation, with one data point per group). In fact, a threefold increase in the size of the group has no discernible impact on PGG contributions in the treatment.

In contrast, final round contributions in the control do seem to decrease as groups grow larger, albeit only at a marginal level of statistical significance (coeff = -0.110, p = 0.069, Table S13 col. 1). When data from both the control and treatment conditions are taken together, we correspondingly observe a positive interaction between a treatment dummy and group size (coeff = 0.095, p = 0.204, Table S13 col. 3), suggesting that our intervention if anything becomes more effective relative to the control as groups becomes larger, although this interaction does not achieve statistical significance (perhaps not surprisingly given that we have only 8 independent observations per condition and thus

the statistical test has little power). Results when considering average PGG contributions over all rounds are qualitatively similar (Table S14).

Table S13: Linear regression model estimating the effect of group size on PGG contributions in the final round of the game. Each session corresponds to one observation, and robust standard errors are used.

	Control	Treatment	Both
Group size	-0.110	-0.015	-0.110
	(0.050)	(0.051)	(0.050)*
1=Treatment			3.521
			(3.077)
1=Treatment X group size			0.095
			(0.071)
Constant	11.125	14.646	11.125
	(2.469)**	(1.836)***	(2.469)***
R^2	0.36	0.01	0.85
N	8	8	16

* p<0.05; ** p<0.01; *** p<0.001

Table S14: Linear regression model estimating the effect of group size on PGG contributions averaged over all rounds of the game. Each session corresponds to one observation, and robust standard errors are used.

	Control	Treatment	Both
Group size	-0.052	-0.011	-0.052
	(0.035)	(0.029)	(0.035)
1=Treatment			-0.318
			(2.003)
1=Treatment X group size			0.041
			(0.046)
Constant	15.030	14.712	15.030
	(1.672)***	(1.103)***	(1.672)***
R^2	0.23	0.02	0.37
N	8	8	16

* p<0.05; ** p<0.01; *** p<0.001

2.5.2 Large-scale PGG with 1,000 players

To further address the question of scalability, we ran an additional experiment with 1,000 participants playing one large PGG. In the treatment condition, participants were able to see the contributions of the player with whom they also played a repeated PD after each round of the PGG. Conversely, in the control condition, participants were not able to see the contributions of their PD partner. However, each player in the control condition saw the contributions of another player who was simultaneously playing the same game with someone else (see SI Section 1 for experimental details).

This design required four participants playing the game simultaneously in two pairs in the control condition, and the decisions between those pairs are not independent. Thus, to account for this interdependence, we cluster standard errors at this "double pairs" level (i.e., four players in two pairs playing the game simultaneously). To keep decision decision times and dropout rates constant between control and treatment, we also required that two pairs of participants played the game simultaneously in the treatment group and thus we also cluster on double pairs.

As before, we predicted that contributions would be lower in control than treatment, and this difference would emerge over time. This is indeed what we found: overall levels of contribution were lower in control than treatment (coeff = -1.456, $p = 0.005$, Table S15 col. 1). Over time, participants in the control condition decreased their contributions (coeff = -0.136, $p < 0.001$, Table S15 col. 2), while contributions remained stable in treatment (coeff = -0.027, $p = 0.429$, Table S15 col. 3). This difference was significant when we combined the data from both conditions (interaction between number of rounds and control dummy, coeff = -0.109, $p = 0.017$, Table S15 col. 4).

Furthermore, we found qualitatively similar results when we include dropout groups in our analysis (Table S15 col. 5).

Table S15: Linear regression model estimating the effect of experimental condition and round on contributions in the large public goods game. Standard errors clustered on "double pairs" (groups of four simultaneous players).

	Combined	Control	Treatment	Interaction	Interaction
1=Control	-1.456<br>(0.514)**			-0.855<br>(0.504)	-0.886<br>(0.431)*
Round		-0.136<br>(0.030)***	-0.027<br>(0.034)	-0.027<br>(0.034)	-0.044<br>(0.036)
1=Control X Round				-0.109<br>(0.046)*	-0.098<br>(0.049)*
Constant	11.912<br>(0.362)***	11.206<br>(0.361)***	12.061<br>(0.354)***	12.061<br>(0.353)***	12.194<br>(0.307)***
Dropouts included	No	No	No	No	Yes
R^2	0.01	0.00	0.00	0.01	0.01
N	10,000	5,000	5,000	10,000	11,620

* $p<0.05$; ** $p<0.01$; *** $p<0.001$

3. Instructions

3.1 Experiment 1

A red box indicates treatment **only**; all else was identical across conditions.

Page 1: (Green box = correct answers to comprehension questions)

SoPHIE

Instructions

Thank you for accepting this HIT!

Make sure your read these instructions carefully but fast – the experiment will start as soon as enough people have read the instructions and passed the comprehension questions!

You have been randomly assigned to a large group of people. Please read the following instructions. All participants receive the same instructions.

As soon as all participants have read these instructions and answered the questions below, the decision-making game will automatically begin.

The game consists of **many rounds**. Each round is identical and consists of **2 stages**:

- Stage 1: Common project with many participants
- Stage 2: Partner interactions with 2 participants (the same partners each round)

(To receive the base pay and be eligible for a bonus, DO NOT leave the study before all rounds have been played.)

Stage 1:

In this stage you interact with **many other participants.**

Each of you is given **20 units** in Stage 1 each round.

You can decide how many units to **keep for yourself** and how many to contribute towards a **common project**. You can contribute between 0 and 20 units.

You **must** make your decision **within 20 seconds**. Otherwise you will be **automatically disqualified** from further participation in the game and will not receive any payment or bonus.

Each unit contributed to the common project is **multiplied by 2** and **distributed equally** among all players in the study.

Your income per round is, thus, the number of **units that you keep plus** the **equal share** of units that you receive **from the common project**.

Here are two **examples**:

Example 1:

- Say there are 100 participants and all 100 participants contribute 20 units to the project.
- Then every participant receives 40 units: 20 units initially received – 20 units contributed + 2 * (100 * 20) / 100 units received from pool

Example 2:

- Say there are 100 participants: 90 participants contribute 20 and 10 participants contribute 0.
- Then the 90 **contributing** participants each earn 36 units:
  20 units initially received – 20 units contributed + 2 * (90 * 20) / 100 units received from pool
- And the 10 **non-contributing** participants each earn 56 units:
  20 units initially – 0 units contributed + 2 * (90 * 20) / 100 units received from pool

Stage 2:

In this stage you **interact individually** with 2 other participants. **Throughout the game, you will interact with the *same* 2 participants in every round.** However, if one of them drops out of the game unexpectedly, he or she will be replaced with another participant.

You will **see how many units both participants contributed** to the project in Stage 1 in this round.

You must decide between two actions (A or B) toward each of the other two participants:

If you **choose A**, then **you** pay **6 units** for the **other participant** to get **18 units**.

If you **choose B**, then **you** pay **0 units** for the **other participant** to get **0 units**.

You **must** make your decision **within 20 seconds**. Otherwise you will be **automatically disqualified** from further participation in the game and will not receive any payment or bonus.

Your income in Stage 2 is the number of **units that you receive** from the other 2 participants' actions towards you **minus** the number of **units that you spend** to increase the other participants' payoff.

Summary Stage:

At the end of Stage 2, you will see a summary of both stages and your combined payoff in this round. Then you will move to the **next round of the game** (consisting of the same two stages).

The game will end after an unknown number of rounds. Your behaviour has **no effect on the number of rounds**. **At the end of the game**, the number of units you accumulated will be converted to US dollars for your bonus payment: **60 units = $0.10**.

You must finish the game to receive your base pay ($3.00) and your bonus payment.

Before the start of the real study, **everyone will play one practice round with the computer. The practice round does not impact your bonus.** Afterwards the real study will begin immediatelY: you will be randomly assigned your two partners, which will remain the same throughout the game.

You will now be asked **comprehension questions** about this game to be eligible to participate:

1) In Stage 1, if 50 participants contribute 20 units, 30 participants contribute 10 units, and 20 participants contribute 0 units to the common project, how many units have been **contributed to the common project in total?**

- ◯ A total of 1000 units.
- ◯ A total of 1300 units.
- ◯ A total of 2000 units.

2) **What happens to the units** once they have been contributed to the common project?

- ◯ They get multiplied by 2 and distributed equally to all 100 participants in the game.
- ◯ They get multiplied by 10 and distributed only to those who contributed to the common project.
- ◯ Nothing happens, at all.

3) **In Stage 2**, which one of the following is a **valid action** you can take towards two other participants?

- ◯ Take away 10 units from the two participants without paying any units.
- ◯ Pay 6 units to increase the payoff of another participant by 18 units.
- ◯ Pay 1 unit and get 1 unit from another participant in return.

Note: The information in the red box was only available in the treatment condition.

Page 2:

SoPHIE

Please wait for the other participants ...

You have been randomly assigned to a large group of people. Please wait until everyone is ready to begin.

This will take **approximately 10 minutes**.

SoPHIE

Please wait for the other participants ...

You have been randomly assigned to a large group of people. Please wait until everyone is ready to begin.

The experiment will start exactly in 02:49.

Pages 3-5 (practice round; each screenshot shows one page)

SoPHIE 0:33

THIS IS A PRACTICE ROUND. This practice round is played with a computer. Your decisions will not impact your bonus. The real study will begin IMMEDIATELY AFTER THIS PRACTICE ROUND ENDS.
During the practice round you are given more time to read and make your decision. You have 40 seconds now but as soon as the real study begins you must make your decision within 20 seconds.

Stage 1

How much do you wish to contribute to the common project? (0 to 20 units)

Your contribution:

Submit ...

SoPHIE 0:12

THIS IS A PRACTICE ROUND. This practice round is played with a computer. Your decisions will not impact your bonus. The real study will begin IMMEDIATELY AFTER THIS PRACTICE ROUND ENDS.
During the practice round you are given more time to read and make your decision. You have 40 seconds now but as soon as the real study begins you must make your decision within 20 seconds.

In this hypothetical round, assume that you have a total of **29.17 units** from Stage 1.

Stage 2

Please decide between two actions (A or B) toward each of the two (hypothetical) partners:

If you **choose A**, then **you** pay **6 units** for the **other participant** to get **18 units**.
If you **choose B**, then **you** pay **0 units** for the **other participant** to get **0 units**.

In the real game you will be matched with two real MTurk participants who make real decisions. **But in the practice round a computer just makes decisions *randomly*:**

		Contribution	Choose your action
YOU →	Player 1:	20	
	Player 2:	20	Option A Option B
	Player 3:	10	Option A Option B

Note: The information in the red box was only available in the treatment condition.

SoPHIE 0:05

THIS IS A PRACTICE ROUND. This practice round is played with a computer. Your decisions will not impact your bonus. The real study will begin IMMEDIATELY AFTER THIS PRACTICE ROUND ENDS.

Summary

In the real game you will be matched with two real MTurk participants who make real decisions. **But in the practice round a computer just makes decisions *randomly*:**

Stage 2 payoff:

Interaction with player 2:

Player 2's action towards you:	Option B	You got **0 units**.
Your action towards player 2:	Option A	You paid **6 units** for player 2 to get 18 units.

Interaction with player 3:

Player 3's action towards you:	Option A	You got **18 units**.
Your action towards player 3:	Option A	You paid **6 units** for player 3 to get 18 units.

Your Stage 2 payoff: **6 units**

In THIS PRACTICE ROUND, you earned the following units:

STAGE 1:	38.17 units
STAGE 2:	6 units
**TOTAL THIS PRACTICE ROUND:**	44.17 units

Continue ...

Pages 6-9 (real game, repeated 20 times; each screenshot shows one page):

SoPHIE 0:14

THIS IS THE REAL GAME. Your decisions will impact your bonus.

Warning: you only have 20 seconds to make a decision!

You have been assigned your two partners for stage 2. **The partners are the same two MTurkers every round.**

Stage 1

Remember: You only have **20 seconds to decide!**

How much do you wish to contribute to the common project? (0 to 20 units)

Your contribution:

Submit ...

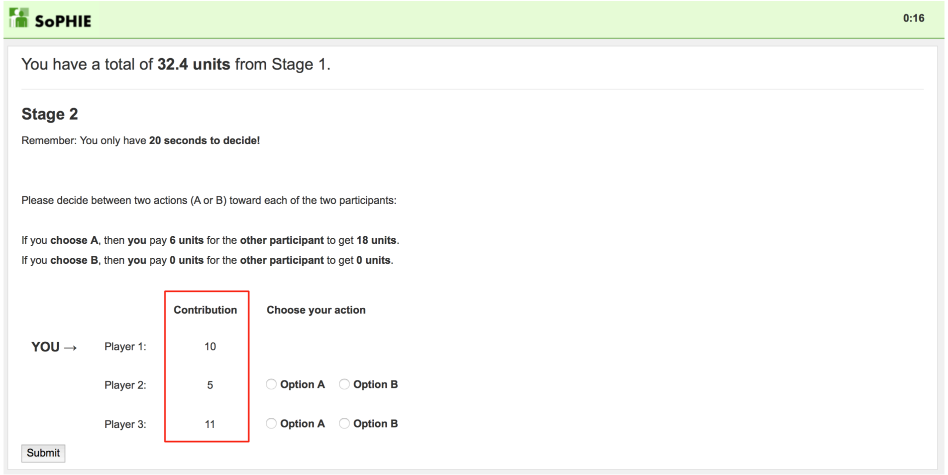

Note: The information in the red box was only available in the treatment condition.

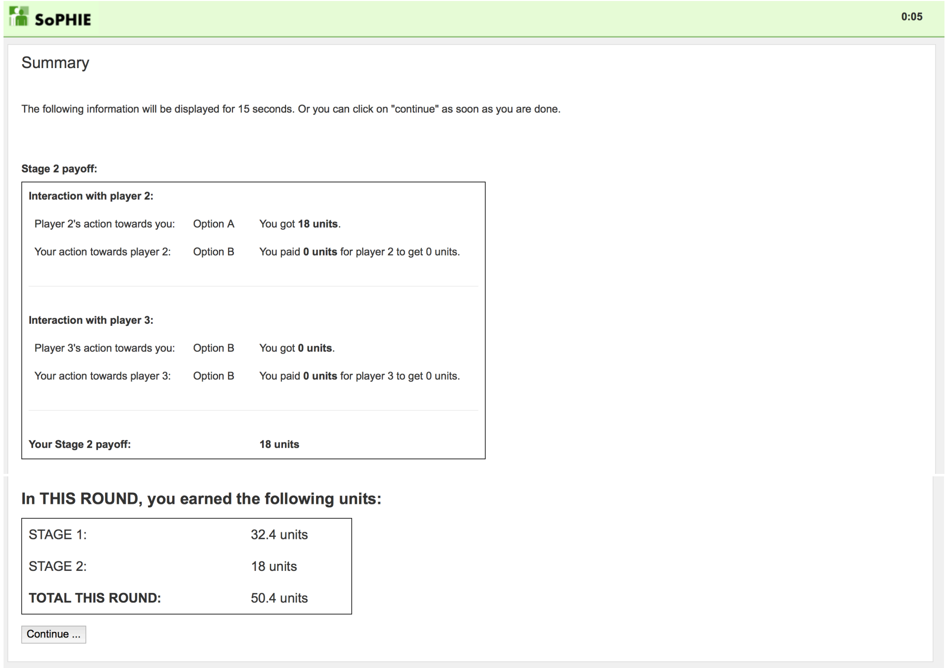

3.2 Experiment 2

3.2.1 Control condition

Page 1: (Green box = correct answers to comprehension questions)

Instructions

Thank you for accepting this HIT!

You have been randomly assigned to a **very large group of 1000 people**. Please read the following instructions.

The game consists of **many rounds**. Each round is identical and consists of **2 stages**:

- Stage 1: Group interaction with 1000 participants.
- Stage 2: Individual interaction with 1 other participant (the same partner each round) who is online at the same time as you.

Note that your decision in Stage 1 will **affect the bonuses of all other 999 people** in your group and **their decisions will affect your bonus**. In addition, your decision in Stage 2 will **affect your partner's bonus and your partner's decision will affect your bonus**.

(To receive the base pay and be eligible for a bonus, DO NOT leave the study before all rounds have been played.)

Stage 1:

In this stage you play a game with **999 other participants.** Each of you is given **20 units** in Stage 1 each round.

You can decide how many units to **keep for yourself** and how many to contribute to a **common project**.

You **must** make your decision **promptly**. Otherwise you will be **automatically disqualified** from further participation in the game and will not receive any payment or bonus.

Each unit contributed to the common project is **doubled**. Once all 1000 people have participated in this study, all doubled units will be **distributed equally** among every single group member, regardless of whether or how much they contributed to the common project.

Thus, for every unit you contribute, you personally lose money but the group as a whole benefits.

Your income from stage 1 is thus the number of **units that you keep plus** an **equal share** of units that you receive at the end of the study **from the common project**.

Here are two **examples**:

Example 1:

- There are 1000 participants in the group. Say, in one round, all 1000 participants contribute 20 units to the project.
- For that round, every participant receives 40 units from Stage 1 in return (which will be paid out only after the end of the study when all 1000 participants have played).

Example 2:

- There are 1000 participants. In one round, 900 participants contribute 20 units and 100 participants contribute 0 units.
- Then the 900 **"contributors"** each earn 36 units in Stage 1 of that round:
  20 units initially – 20 units contributed + 2 * (900 * 20) / 1000 units from common project
- While the 100 **"non-contributors"** each earn 56 units in Stage 1 of that round:
  20 units initially – 0 units contributed + 2 * (900 * 20) / 1000 units from common project

Stage 2:

In this stage you **interact individually** with 1 other participant of the 1000-participants group. **You will be Player A and the participant you will interact with is Player B.**

Throughout the game, you will interact with the **same participant as Player B** in every round. Player B has the same options as you in Stage 2.

You will ***not*** **find out how many units Player B contributed** to the common project in Stage 1.

There will also be **a third participant called Player C**: Each round, you will **see how many units Player C contributed** to the common project in Stage 1. **Note that you will *not* interact with Player C individually in Stage 2.**

For your individual interaction with Player B in Stage 2, you receive an endowment of 12 units each round. You must decide between two actions (Y or Z) **toward Player B**:

If you **choose Y**, then **you** pay **12 units** for **Player B** to get **36 units**.

If you **choose Z**, then **you** pay **0 units** for **Player B** to get **0 units**.

You **must** make your decision **promptly**. Otherwise you will be **automatically disqualified** from further participation in the game and will not receive any payment or bonus.

Your income in Stage 2 is **12 units plus** the number of **units that you receive** from Player B's action towards you **minus** the number of **units that you spend** to increase Player B's payoff.

Summary Stage:

At the end of Stage 2, you will see a summary of your actions in both stages. Then you will move to the **next round of the game** (consisting of the same two stages). The game will end after an unknown number of rounds. Your behaviour has **no effect on the number of rounds**.

After all rounds have been played, the number of units you accumulated will be converted to US dollars for your bonus payment. **You will receive your bonus payment in two installments**: (1) first you will be paid your bonus from Stage 2 (typically within 24 hours); and (2) when all 1000 participants have completed the study, you will receive your bonus from Stage 1 (probably within 2-3 weeks). The conversion rate is **160 units = $0.10**.

You must finish the game to receive your base pay ($1.00) and your bonus payment.

You will now be asked **comprehension questions** about this game to be eligible to participate:

1) In Stage 1, how many units should you invest to maximize the benefit to the group as whole?

- Contributing 0 units.
- Contributing 20 units.
- Contributing 5 units.

2) In Stage 1, how many units should you invest to maximize your own payoff?

- Contributing 0 units.
- Contributing 20 units.
- Contributing 15 units.

3) In Stage 2, what information from Stage 1 will you see?

- I will see how many units the participant who I interact with (Player B) contributed in Stage 1.
- I will see how many units another participant who I don't interact with (Player C) contributed in Stage 1.
- I will only see how many units I contributed in Stage 1.

4) Which one of the following is a valid action you can take in Stage 2?

- Take away 10 units from the two random participants without paying any units.
- Pay 12 units to increase the payoff of Player B by 36 units.
- Pay 1 unit and get 1 unit from a random participant in return.

5) When will you receive your bonus?

- Right after I submit the HIT.
- There will be no bonus in this HIT.
- The bonus will be paid in 2 separate installments: I will receive one bonus payment soon after submitting the HIT and I will receive another bonus payment in a few weeks' time.

Pages 2-4 (repeated 10 times; each screenshot shows one page):

Time left: 25 seconds

Your endowment in Stage 1 is 20 units.

Please decide now how many units you want to contribute to the large common project with a total of 1000 participants.

(Type a value between 0 and 20.)

Submit ...

Time left: 24 seconds

STAGE 2:

Your endowment in Stage 2 is 12 units.

Below you can choose between **two options (Y or Z)** **towards Player B**. You always interact with the same participant (Player B) over all rounds. Player B has the same options to choose from towards you.

If you **choose Option Y**, then **you** pay **12 units** for **Player B** to get **36 units**.

If you **choose Option Z**, then **you** pay **0 units** for **Player B** to get **0 units**.

Please decide now which action to take **towards Player B**:

	PLAYER	STAGE 1 CONTRIBUTION	OPTIONS
**YOU →**	Player A:	20 units	**← YOU**
	Player B:	10 units	Option Y<br>Option Z

Submit ...

Your decisions in this round:

STAGE 1:

You contributed to common project:	**20** units
Units from the common project to you:	Will be calculated **at the end of the study**

STAGE 2:

You gave to your partner:	**0 units** (Option Z)
Your partner gave to you:	**36 units** (Option Y)

Continue ...

3.2.2 Treatment condition

Page 1: (Green box = correct answers to comprehension questions)

Instructions

Thank you for accepting this HIT!

You have been randomly assigned to a **very large group of 1000 people**. Please read the following instructions.

The game consists of **many rounds**. Each round is identical and consists of **2 stages**:

- Stage 1: Group interaction with 1000 participants.
- Stage 2: Individual interaction with 1 other participant (the same partner each round) who is online at the same time as you.

Note that your decision in Stage 1 will **affect the bonuses of all other 999 people** in your group and **their decisions will affect your bonus**. In addition, your decision in Stage 2 will **affect your partner's bonus and your partner's decision will affect your bonus**.

(To receive the base pay and be eligible for a bonus, DO NOT leave the study before all rounds have been played.)

Stage 1:

In this stage you play a game with **999 other participants.** Each of you is given **20 units** in Stage 1 each round.

You can decide how many units to **keep for yourself** and how many to contribute to a **common project**.

You **must** make your decision **promptly**. Otherwise you will be **automatically disqualified** from further participation in the game and will not receive any payment or bonus.

Each unit contributed to the common project is **doubled**. Once all 1000 people have participated in this study, all doubled units will be **distributed equally** among every single group member, regardless of whether or how much they contributed to the common project.

Thus, for every unit you contribute, you personally lose money but the group as a whole benefits.

Your income from stage 1 is thus the number of **units that you keep plus** an **equal share** of units that you receive at the end of the study **from the common project**.

Here are two **examples**:

Example 1:

- There are 1000 participants in the group. Say, in one round, all 1000 participants contribute 20 units to the project.
- For that round, every participant receives 40 units from Stage 1 in return (which will be paid out only after the end of the study when all 1000 participants have played).

Example 2:

- There are 1000 participants. In one round, 900 participants contribute 20 units and 100 participants contribute 0 units.
- Then the 900 **"contributors"** each earn 36 units in Stage 1 of that round:
  20 units initially – 20 units contributed + 2 * (900 * 20) / 1000 units from common project
- While the 100 **"non-contributors"** each earn 56 units in Stage 1 of that round:
  20 units initially – 0 units contributed + 2 * (900 * 20) / 1000 units from common project

Stage 2:

In this stage you **interact individually** with 1 other participant of the 1000-participants group. **You will be Player A and the participant you will interact with is Player B.**

Throughout the game, you will interact with the **same participant as Player B** in every round. Player B has the same options as you in Stage 2.

Each round, you will **see how many units Player B contributed** to the common project in Stage 1. **Player B will see how many units you contributed** to the common project in Stage 1.

For your individual interaction with Player B in Stage 2, you receive an endowment of 12 units each round. You must decide between two actions (Y or Z) **toward Player B**:

If you **choose Y**, then **you** pay **12 units** for **Player B** to get **36 units**.

If you **choose Z**, then **you** pay **0 units** for **Player B** to get **0 units**.

You **must** make your decision **promptly**. Otherwise you will be **automatically disqualified** from further participation in the game and will not receive any payment or bonus.

Your income in Stage 2 is **12 units plus** the number of **units that you receive** from Player B's action towards you **minus** the number of **units that you spend** to increase Player B's payoff.

Summary Stage:

At the end of Stage 2, you will see a summary of your actions in both stages. Then you will move to the **next round of the game** (consisting of the same two stages). The game will end after an unknown number of rounds. Your behaviour has **no effect on the number of rounds**.

After all rounds have been played, the number of units you accumulated will be converted to US dollars for your bonus payment. **You will receive your bonus payment in two installments**: (1) first you will be paid your bonus from Stage 2 (typically within 24 hours); and (2) when all 1000 participants have completed the study, you will receive your bonus from Stage 1 (probably within 2-3 weeks). The conversion rate is **160 units = $0.10**.

You must finish the game to receive your base pay ($1.00) and your bonus payment.

You will now be asked **comprehension questions** about this game to be eligible to participate:

1) In Stage 1, how many units should you invest to maximize the benefit to the group as whole?

- Contributing 0 units.
- Contributing 20 units.
- Contributing 5 units.

2) In Stage 1, how many units should you invest to maximize your own payoff?

- Contributing 0 units.
- Contributing 20 units.
- Contributing 15 units.

3) In Stage 2, what information from Stage 1 will you see?

- I will see how many units the participant who I interact with (Player B) contributed in Stage 1.
- I will see how many units another participant who I don't interact with (Player C) contributed in Stage 1.
- I will only see how many units I contributed in Stage 1.

4) Which one of the following is a valid action you can take in Stage 2?

- Take away 10 units from the two random participants without paying any units.
- Pay 12 units to increase the payoff of Player B by 36 units.
- Pay 1 unit and get 1 unit from a random participant in return.

5) When will you receive your bonus?

- Right after I submit the HIT.
- There will be no bonus in this HIT.
- The bonus will be paid in 2 separate installments: I will receive one bonus payment soon after submitting the HIT and I will receive another bonus payment in a few weeks' time.

Pages 2-4 (repeated 10 times; each screenshot shows one page):

Time left: 25 seconds

Your endowment in Stage 1 is 20 units.

Please decide now how many units you want to contribute to the large common project with a total of 1000 participants.

(Type a value between 0 and 20.)

Submit ...

Time left: 23 seconds

STAGE 2:

Your endowment in Stage 2 is 12 units.

Below you can choose between **two options (Y or Z) towards Player B**. You always interact with the same participant (Player B) over all rounds. Player B has the same options to choose from towards you.

If you **choose Option Y**, then **you** pay **12 units** for **Player B** to get **36 units**.

If you **choose Option Z**, then **you** pay **0 units** for **Player B** to get **0 units**.

Please decide now which action to take **towards Player B**:

	PLAYER	STAGE 1 CONTRIBUTION	OPTIONS
**YOU →**	Player A:	20 units	← **YOU**
	Player B:	Not shown	○ Option Y<br>○ Option Z
	Player C:	10 units	No options

Submit ...

Your decisions in this round:

STAGE 1:

You contributed to common project:	**20** units
Units from the common project to you:	Will be calculated<br>**at the end of the study**

STAGE 2:

You gave to your partner:	**0 units** (Option Z)
Your partner gave to you:	**0 units** (Option Z)

Continue ...

References

1. Rand DG, Greene JD, Nowak MA (2012) Spontaneous giving and calculated greed. *Nature* 489(7416):427–430.

2. Amir O, Rand DG, Gal YK, Gal YK (2012) Economic Games on the Internet: The Effect of $1 Stakes. *PLoS ONE* 7(2):e31461.

3. Horton JJ, Rand DG, Zeckhauser RJ (2011) The online laboratory: conducting experiments in a real labor market. *Exp Econ* 14(3):399–425.

4. Berinsky AJ, Huber GA, Lenz GS (2012) Evaluating online labor markets for experimental research: Amazon. com's Mechanical Turk. *Political Analysis* 20:351–368.

5. Hendriks A (2012) SoPHIE - Software Platform for Human Interaction Experiments. *Working Paper*.

6. Rand DG, Arbesman S, Christakis NA (2011) Dynamic social networks promote cooperation in experiments with humans. *Proceedings of the National Academy of Sciences* 108(48):19193–19198.

7. Rand DG, Nowak MA, Fowler JH, Christakis NA (2014) Static network structure can stabilize human cooperation. *Proceedings of the National Academy of Sciences* 111(48):17093–17098.

8. Andreoni J (1988) Why free ride? *J Public Econ* 37(3):291–304.

9. Croson R (1996) Partners and strangers revisited. *Economics Letters* 53(1):25–32.

10. Rand DG, Dreber A, Ellingsen T, Fudenberg D, Nowak MA (2009) Positive interactions promote public cooperation. *Science* 325:1272–1275.

11. Fischbacher U, Gächter S, Fehr E (2001) Are people conditionally cooperative? Evidence from a public goods experiment. *Economics Letters* 71(3):397–404.
